# Experience matters for robotic assistance: an analysis of case data

**DOI:** 10.1007/s11701-023-01677-w

**Published:** 2023-07-14

**Authors:** Riley Brian, Daniel Oh, Kelli Ann Ifuku, Ankit Sarin, Patricia O’Sullivan, Hueylan Chern

**Affiliations:** 1 Department of Surgery, University of California San Francisco, San Francisco, CA USA; 2 Department of Surgery, University of Southern California, Los Angeles, CA USA; 3 Intuitive Surgical, Sunnyvale, CA USA; 4 Department of Surgery, University of California Davis, Sacramento, CA USA

**Keywords:** Robotic assistance, Bedside assistance, Learning curve, Resident training

## Abstract

Many robotic procedures require active participation by assistants. Most prior work on assistants’ effect on outcomes has been limited in procedural focus and scope, with studies reporting differing results. Knowing how assistant experience affects operating room time could inform operating room case scheduling and provide an impetus for additional assistant training. As such, this retrospective cohort study aimed to determine the association between assistant experience and operating room time for 2291 robotic-assisted operations performed from 2016 to 2022 at our institution. Linear regression showed a significant association between the presence of a junior resident and increased case length differential with an increase of 26.9 min (*p* = 0.01). There were no significant associations between the presence of a senior resident (*p* = 0.52), presence of a fellow (*p* = 0.20), or presence of a physician assistant (*p* = 0.43) and case length differential. The finding of increased operating room time in the presence of a junior resident during robotic cases supports consideration of the adoption of formal assistant training programs for residents to improve efficiency.

## Introduction

Educational interventions in robotic surgery often focus on the surgeon at the console. However, many robotic procedures require active participation by additional surgical team members. Assistants can be junior residents, senior residents, fellows, physician assistants (PAs), and other members of the operative team [1]. In some cases, the role of the assistant is minimal and limited to bedside instrument exchange, camera cleaning, and port site closure. In other operations, the assistant must provide adept laparoscopic assistance and understand the flow and sequence of the operation at an advanced level [2]. The assistant’s experience—whether limited as a junior resident or extensive as a fellow—has been posited as important to the success of robotic surgery [3].

Bedside assistance is frequently described as a stepping stone on the path to console surgeon [4–6]. Prior work has acknowledged the multiple skills required as an assistant, and learning curve analysis has suggested that 10–36 cases are required for proficient bedside assistance [2, 7]. However, the actual training of assistants varies [3, 6]. To expedite assistant education, some authors have piloted courses focusing on a combination of didactic and psychomotor training [8, 9]. Another simulation-based report found that an augmented reality intervention with 3D vision decreased the time and errors in performing complex bedside tasks [10]. To provide feedback and assess bedside assistants, one group developed validity evidence for a modified Objective Structured Assessment of Technical Skills (OSATS) [11]. Nonetheless, there is no consensus on a standardized assistant training or assessment program; thus, the experience among assistants differs widely [8].

The effect of operative assistant experience on outcomes has been widely studied in many settings, with different results by procedure, institution, and publication [3, 12–23]. Most of the existing literature on the effect of the assistant in robotic surgery is limited to robotic-assisted prostatectomy and nephrectomy. Given the small samples, limited scopes, and heterogeneous results of prior studies, the importance of experience for assistants remains an unanswered question.

As such, this study aimed to determine the association between assistant experience and operating room time for all robotic-assisted operations performed at our institution. We hypothesized that the presence of more experienced assistants would be associated with shorter operating room time. This information could inform operating room case scheduling and provide an impetus for additional assistant training.

## Methods

### Design

This was a correlational study assessing predictor variables’ association with the outcome variable of case length differential, which was defined as the difference between the actual operating room (OR) time and the scheduled OR time, including surgical and anesthesia time. Predictor variables were classified as patient related or surgical team related. Patient-related predictor variables comprised patient body mass index (BMI), patient pre-operative opioid use, patient smoking status, and patient American Society of Anesthesiologists (ASA) physical status classification. These patient-related predictor variables were chosen given that prior work has also assessed their possible contribution to peri-operative and OR time [24–27]. Patient-related predictor variable data were not available for all cases. Surgical team-related predictor variables included the number of junior residents, the number of senior residents, the number of fellows, and PA presence. Junior residents were defined as post-graduate year 1 and 2, while senior residents were defined as post-graduate year 3 and greater. Surgical team-related predictor variable data were available for every case. The outcome variable was case length differential. The single outcome variable was pre-specified prior to initiating the analysis.

### Setting

This study was conducted at a single tertiary-level academic medical center. At this medical center, the assistant may be a resident, fellow, physician assistant, or there may be no assistant present. Experience among assistants varies significantly with no standardized assistant requirements. Surgical technologists do not dock the robot or exchange instruments at our institution.

### Data collection

Data were collected retrospectively from the electronic medical record and operating room archives. All patients who underwent a Da Vinci robotic-assisted operation from January, 2016 to August, 2022 were included (Intuitive Surgical. Sunnyvale, CA, USA). Data included date of surgery, procedure, primary surgeon, all surgeons, PA presence, scheduled start time, scheduled end time, actual start time, actual end time, patient demographics, patient BMI, patient pre-operative opioid use, patient smoking status, and patient ASA physical status classification. All surgeons were subclassified as junior residents, senior residents, fellows, or attendings.

### Data analysis

Descriptive data were generated. Two linear regression was performed: one to assess the impact of the patient-related predictor variables on the outcome variable of case length differential and a second to assess the impact of surgical team-related predictor variables on the outcome variable of case length differential. Stata/IC V.16.1 for Mac (StataCorp. College Station, TX, USA) was used for data analysis.

### Ethical approval

This study was approved by our Institutional Review Board (IRB22-37360).

## Results

During the study period, 2291 robotic-assisted cases were performed and included in the study. Robotic-assisted prostatectomy was the most commonly performed procedure (28.9%; 661 of 2291), followed by robotic-assisted hysterectomy (13.1%; 301 of 2291), and robotic-assisted inguinal hernia repair (9.9%; 227 of 2291).

Of the 2291 cases, 751 contained data on patient-related predictor variables. Among those cases, mean BMI was 26.9 (SD 9.0) and the median ASA physical status classification was 2 (IQR 2–3). Almost all patients (98.1%) were non-smokers and most patients (89.6%) were not using opioids prior to surgery. All 2291 cases contained data on surgical team-related predictor variables. Of these, 1094 cases (47.8%) involved trainees and 126 cases (5.5%) involved PAs (Table 1). One hundred forty-six cases (6.4%) involved more than one trainee. Median actual OR time was 407 min (IQR 324–502). Median scheduled OR time was 400 min (IQR 335–470). The median case length differential was 8 min (IQR − 42 to 81).

**Table 1:** Team member involvement in cases

Case type	Total number of cases *n*	Junior resident present *n* (%)	Senior resident present *n* (%)	Fellow present *n* (%)	Physician assistant present *n* (%)	Multiple trainees present *n* (%)
All cases	2291	138 (6)	336 (15)	674 (29)	126 (6)	146 (6)
Prostatectomy	661	1 (0)	28 (4)	268 (41)	21 (3)	16 (2)
Hysterectomy	301	11 (4)	91 (30)	79 (26)	33 (11)	33 (11)
Inguinal hernia repair	227	48 (21)	42 (19)	32 (14)	5 (2)	16 (7)

Multiple types of team members were involved in cases, which varied among the three most commonly performed cases

**Fig. 1:**
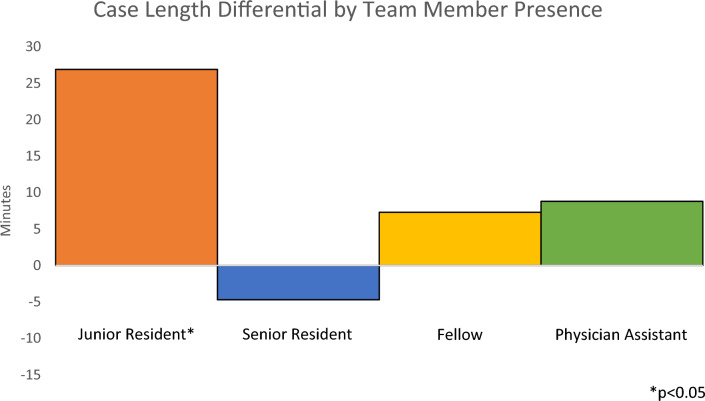
Case length differential by team member presence. The presence of a junior resident was significantly associated (*p* = 0.01) with increased case length differential (actual minus scheduled operating room time) during robotic-assisted procedures

Linear regressions were performed to assess the impact of the patient-related and surgical team-related predictor variables on the outcome variable of case length differential (Table 2). Regression assessing the impact of patient-related predictor variables showed no significant association between BMI (*p* = 0.98), ASA physical status classification (*p* = 0.26), smoking (*p* = 0.34), or pre-operative opioid use (*p* = 0.29) and case length differential. Regression assessing the impact of surgical team-related predictor variables showed a significant association between the presence of a junior resident and increased case length differential with an increase of 26.9 min (*p* = 0.01) (Table 3). There was no significant association between the presence of a senior resident (*p* = 0.52), presence of a fellow (*p* = 0.20), or presence of a PA (*p* = 0.43) and case length differential (Fig. 1).

**Table 2:** Association between patient-related predictor variables and outcome variable

Variable	Coefficient	Standard error	*t*-statistic	*p* value
BMI	− 0.01	0.52	− 0.02	0.98
ASA	8.95	7.96	1.12	0.26
Smoking	31.99	33.49	0.96	0.34
Pre-operative opioid use	15.89	15.07	1.05	0.29
*R*-squared	0.0052			
Adjusted *R*-squared	− 0.0001			

No patient-related predictor variables were significantly associated with the outcome variable of case length differential (actual minus scheduled operating room time) by linear regression

*BMI* body mass index, *ASA* American Society of Anesthesiologists physical status classification

**Table 3:** Association between surgical team-related predictor variables and outcome variable

Variable	Coefficient	Standard error	*t*-statistic	*p* value
Presence of junior resident	26.94	10.64	2.53	0.01
Presence of senior resident	− 4.65	7.27	− 0.64	0.52
Presence of fellow	7.31	5.69	1.29	0.20
Presence of physician assistant	8.76	11.06	0.79	0.43
*R*-squared	0.0038			
Adjusted *R*-squared	0.0020			

The presence of a junior resident was significantly associated with the outcome variable of case length differential (actual minus scheduled operating room time) by linear regression

Linear regression assessing the impact of surgical team-related predictor variables was repeated for cases without any missing patient-related predictor variables, yielding similar results. This regression was also repeated after removing cases in which more than one trainee was present, given that the role of the bedside assistant was less clear in such cases. This repeated analysis also yielded similar results.

## Discussion

In summary, this correlational study demonstrated that the presence of a junior resident during a robotic-assisted procedure was associated with increased OR time by approximately 27 min for 2291 robotic cases at a single institution from 2016 to 2022. The presence of other team members was not significantly associated with changes in OR time.

These findings build on prior work that has also evaluated the experience of the assistant in robotic surgery. Indeed, one previous study assessed the effect of assistant experience in 170 cases of robotic-assisted radical prostatectomy and concluded that an expert assistant (i.e., assistants who had completed formal training) was associated with shorter length of stay, less estimated blood loss, and lower positive margin rate [3]. Another study analyzed case length for 222 assistants in urology by comparing their early and late cases, and found a modest decrease in case length with experience [19]. In contrast, three other studies in prostatectomy and nephrectomy involving a combined 636 patients showed no effect on patient outcomes or case length based on the experience of the assistant [18, 20, 21]. Studies of the impact of assistants in 191 robotic-assisted thyroid operations and 105 robotic-assisted Nissen fundoplications came to conflicting results [22, 23]. Our work adds to this literature with a much larger sample size of real-world, diverse cases in multiple specialties with various types of assistants.

The rapid adoption of robotic surgery has required shifting roles and educational changes, with a consensus assistant training pathway yet to materialize. Unfortunately, assistants frequently lack significant experience and learn in unstructured ways [3, 18, 28]. However, studies repeatedly show that formal training with simulation improves operative performance and efficiency in surgery [29]. As such, our finding of increased OR time in the presence of a junior resident during robotic cases provides additional support for the adoption of formal assistant training programs to improve efficiency. Structured education for junior residents may be a mechanism by which to decrease the OR time discrepancy that we observed. Future studies should assess the impact of such formal training on case length in diverse robotic settings. Additional research could investigate the effect of participant role on more patient-centered outcomes, such as length of hospital stay or complications.

This study has a number of limitations. Most notably, this is a retrospective review and we cannot be certain of the responsibilities of participants in each case. While it is very likely that the non-attending member of the operating team acted as the assistant, it is possible that this did not occur in certain instances. However, repeating the analysis for cases with more role uncertainty (i.e., in which more than one trainee was present and thus one trainee may have been at the console) yielded similar results. It is also possible that attendings might request a dedicated assistant for cases they expect to be complex, thus confounding analysis of the effect of another participant. However, our finding with regard to case length differential was limited to junior resident presence. Furthermore, we evaluated a very diverse collection of procedures, and while this expands on previously reported studies that focused on small numbers of homogenous cases, this may introduce uncontrolled variables into the analysis. Clearly, many other factors play large roles in case length differential. Nonetheless, it was notable that the increased OR time associated with a junior resident of about 27 min was substantially greater than the median case length differential of 8 min. Finally, this is a single institution study at a large academic medical center, and the experiences of participants may differ elsewhere.

Overall, we found that the presence of a junior resident during robotic-assisted procedures was associated with increased OR time. Focused education for robotic assistant skills may be warranted for junior trainees to improve their performance and efficiency during robotic cases.

## Data Availability

Data are available upon reasonable request.
